# Design and evaluation of an embedded automation system for optimized cut-shape placement on coconut shells in sustainable key tag manufacturing

**DOI:** 10.1371/journal.pone.0345089

**Published:** 2026-04-06

**Authors:** Saramge Dona Thakshila Roshani, Tharaga Sharmilan

**Affiliations:** Department of Applied Computing, Faculty of Computing and Technology, University of Kelaniya, Kelaniya, Sri Lanka; University of Sharjah, UNITED ARAB EMIRATES

## Abstract

**Purpose:**

This paper presents the design and evaluation of a novel embedded automation system for optimized cut-shape placement on coconut shells in sustainable key tag manufacturing. The system addresses inefficiencies in manual marking by improving speed, accuracy, and material utilization, while ensuring affordability and suitability for decentralized artisanal contexts.

**Design/methodology/approach:**

The system integrates a Raspberry Pi 4, VL53L0X Time-of-Flight sensor, Pi-Camera, MG995 servos, and a low-power laser module, orchestrated through a Python-based finite-state machine (FSM). A lightweight CNN model deployed via TensorFlow Lite enables real-time classification of shell geometries. Comparative experiments were conducted on 20 coconut shells under both manual and automated marking conditions. Evaluation metrics included marking time, accuracy of placement, material utilization, and productive yield.

**Findings:**

The proposed system demonstrated significant improvements over manual marking: marking time was reduced by 26.0%, placement accuracy improved by 79.4%, and material utilization increased by 38.4%. The average number of usable cuts per shell increased from 2.85 (manual) to 3.89 (automated), representing a 36.5% gain in productive yield. All improvements were statistically significant (p < 0.05).

**Originality:**

This is the first known application of a low-cost, edge-AI-enabled embedded system combining ToF sensing, CNN-based shape classification, and adaptive actuation for real-time marking on irregular natural materials. The system dynamically adapts to geometric variability in biodegradable substrates, an area underserved by conventional automation solutions.

**Practical implications:**

The system is cost-effective (<$132), lightweight (<2.5 kg), and fully modular, making it suitable for deployment in SMEs, rural micro-factories, and vocational training centers.

**Social implications:**

By augmenting artisanal labor rather than replacing it, the system supports inclusive digital transformation in low-resource environments and contributes to SDGs 8, 9, and 12. It is also generalizable to other natural materials, supporting broader applications in sustainable, circular manufacturing.

## 1 Introduction

The increasing global emphasis on sustainable and circular production has intensified the search for eco-friendly materials and decentralized manufacturing practices. Among various bio-based resources, coconut shells, a by-product of the coconut industry, have garnered attention for their high lignocellulosic content, structural hardness, and biodegradability. These properties make them ideal for decorative and functional applications such as key tags, jewellery, and homeware. Their utilization supports sustainable waste valorization, promotes bioeconomy practices, and reduces dependence on petroleum-based composites.

Despite these material advantages, manual processing of coconut shells remains a bottleneck in small-scale and rural production units. In particular, the cut-shape marking and placement process still relies heavily on human visual judgment and artisanal skill, leading to significant inconsistency in quality, material waste, and extended production time. Furthermore, repetitive manual labor in these settings can cause physical strain and limit scalability.

Existing industrial automation systems, optimized for regular, synthetic substrates, struggle with irregular natural materials like coconut shells. These systems are often bulky, expensive, and require centralized computing infrastructure, rendering them unsuitable for resource-constrained and artisanal environments. Their lack of adaptability also makes them incompatible with decentralized, low-volume manufacturing typical of rural economies [1,2].

To address these limitations, this study proposes a low-cost, embedded automation system for intelligent cut-shape placement on coconut shells. The system integrates non-contact visual sensing, lightweight edge AI models, and servo-based actuation to enable real-time, high-precision marking. Unlike traditional automation platforms, the proposed system is compact, modular, and cloud-independent, making it suitable for deployment in community-scale production networks. The core objectives of this research are:

1. To design and prototype an embedded vision and actuation system tailored for coconut shell geometry,
2. To optimize material utilization and reduce production variance through AI-based surface mapping and placement algorithms,
3. To evaluate system performance against traditional manual marking in terms of material efficiency, repeatability, and processing time,
4. To assess the system’s feasibility for integration into sustainable and community-centric manufacturing networks.

This study advances the current state-of-the-art in several key ways. Unlike existing automation systems designed for uniform, synthetic substrates, the proposed platform demonstrates a 79.4% improvement in placement accuracy, 38.36% increase in material utilization, and 36.5% gain in productive yield, quantitatively outperforming prior shape-marking approaches on irregular natural materials. The use of a lightweight, edge-deployable CNN classifier coupled with real-time actuation based on ToF sensing presents a novel integration not previously addressed in low-cost, embedded contexts. These enhancements mark a substantial step beyond previous static or manually aided systems and set the foundation for scalable, sustainable, AI-assisted artisanal production.

## 2 Background and related work

Building upon the foundational need for sustainable, decentralized production articulated in Section 1, this section critically examines prior efforts to mechanize natural-material processing, particularly coconut shells, and highlights technological gaps that this study addresses. Coconut shells possess high lignocellulosic strength, thermal resistance, and aesthetic texture, making them ideal for upcycled consumer goods [2–8]. Nevertheless, their integration into streamlined manufacturing pipelines is hindered by manual production bottlenecks, especially in small-scale industries where tasks like cut-shape placement still rely on artisanal skill and imprecise visual heuristics.

### 2.1 Mechanization in artisanal manufacturing

Mechanization of traditional crafts poses both technical limitations and social disruption risks. Many rural manufacturing hubs lack the infrastructure to deploy high-end automation, necessitating cost-effective and adaptable embedded systems. Low-cost programmable platforms have been successfully used in agricultural robotics and traditional weaving systems, showing potential for adaptation to coconut shell workflows. However, current implementations rarely address the geometric variability inherent in natural materials. While few developed robotic drilling solutions for woodcrafts, shape-aware actuation for surface-level marking remains unaddressed in the literature [2–6].

### 2.2 Non-contact sensing for irregular materials

Due to the irregular, semi-spherical geometry of coconut shells, traditional contact-based profiling can lead to alignment errors and surface degradation. Advances in Time-of-Flight (ToF) and structured-light 3D sensors now allow precise non-contact depth mapping on complex organic surfaces. For instance, low-cost optical triangulation and stereo vision techniques have demonstrated sub-millimetre accuracy for surface reconstruction, making them suitable for marking and engraving applications on non-planar surfaces.

### 2.3 Embedded AI and edge computing in low-resource environments

Recent advances in edge AI have enabled deep learning models to operate directly on microcontrollers and single-board computers, eliminating reliance on cloud-based computation. Frameworks such as TensorFlow Lite and TinyML facilitate real-time inference using quantized convolutional neural networks (CNNs) on devices like the Raspberry Pi and Jetson Nano. For instance, TinyML has been used for edge-deployed vision tasks in agriculture, food safety, and defect detection, suggesting its potential for real-time surface classification and placement optimization in natural material domains [2–4].

Several recent works have also emphasized the effectiveness of deep learning in enabling responsive, low-cost systems across diverse domains. For example, deep learning has been used to develop cost-effective assistive robots for autism therapy, semantic mapping solutions using monocular vision in mobile robots, and grain quality assessment systems leveraging affordable sensors. While their application areas differ, these studies support a broader trend of embedding lightweight AI models into constrained environments, closely aligned with our approach of deploying CNN-based classification and real-time actuation for sustainable fabrication on natural materials [9–12].

### 2.4 Low-cost actuation and shape-aware marking

Precision actuation for marking on curved surfaces requires the coordination of sensing and responsive motion control. Servos and stepper motors have been widely used in low-cost fabrication systems such as laser engraving on wood and bamboo. More recently, AI-guided engraving systems have been implemented using adaptive toolpaths generated by embedded vision systems. These examples show the promise of compact, intelligent systems, though specific integration with irregular biomaterials like coconut shells remains underexplored.

### 2.5 Gaps and research direction

While significant progress has been made in vision-based sensing, embedded AI, and low-cost actuation technologies, their integration into a unified system for handling biodegradable, irregular substrates like coconut shells is largely absent [2]. Most existing systems are either cost-prohibitive or only applicable to uniform, planar materials. No existing solution has combined real-time ToF sensing, surface-aware CNN-based classification, and embedded actuation for shape placement optimization on coconut shells.

This study fills that gap by proposing a modular, edge-AI-enabled automation system that couples depth sensing with cut-path optimization and adaptive actuation. By doing so, it aims to enable intelligent, decentralized production suited for rural and small-scale sustainable manufacturing environments.

## 3 System design and architecture for embedded automation in natural material processing

**Fig 1:**
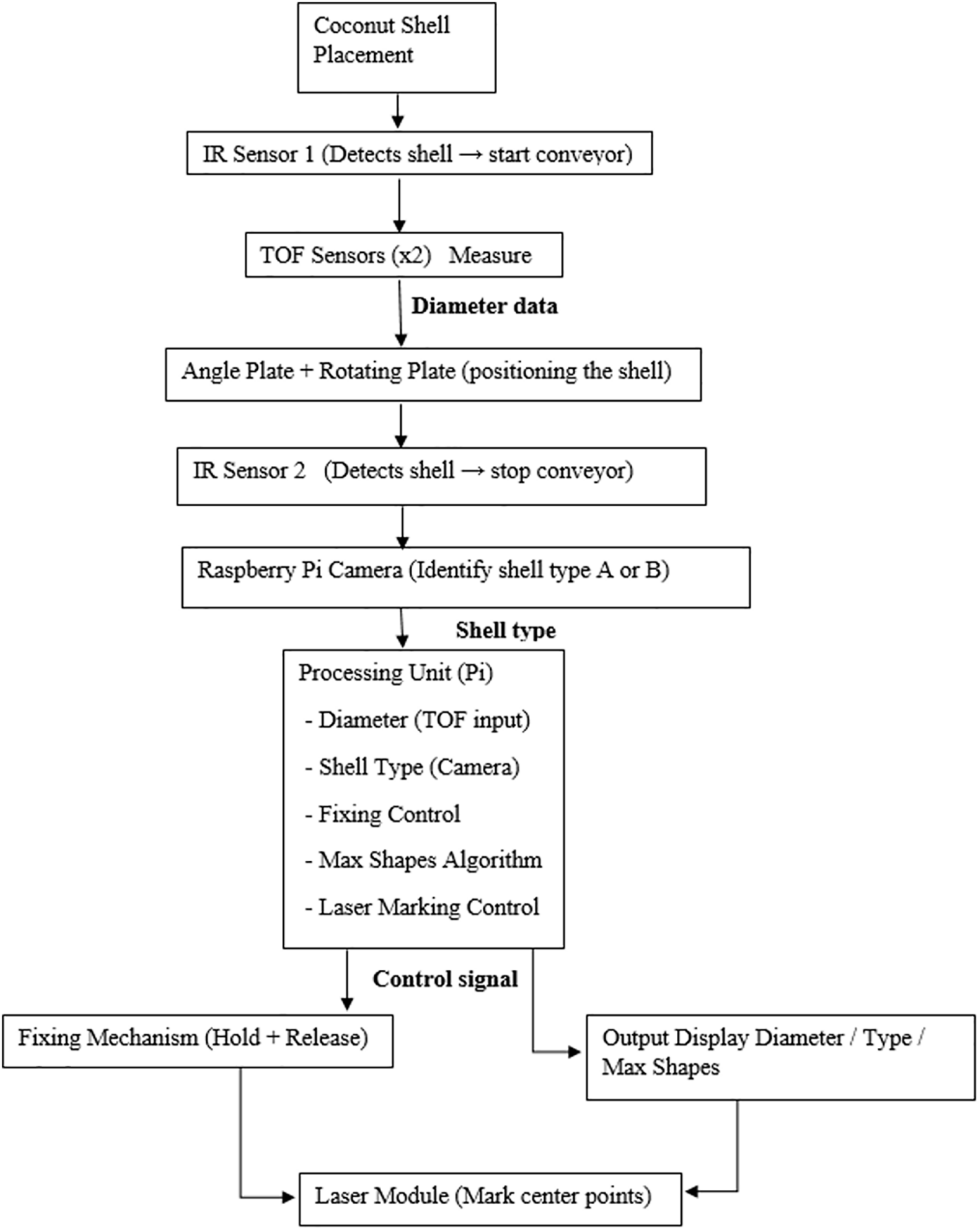
System architecture diagram illustrating the integration of sensing, AI-driven classification, servo-based actuation, laser marking, and FSM control for adaptive cut-shape placement on coconut shells.

In response to the technical limitations identified in Section 2, this research proposes a compact, modular embedded system designed to automate cut-shape placement on coconut shells. The system is optimized for decentralized, small-scale manufacturing environments, emphasizing low cost, adaptability, and real-time processing. The architecture comprises five interlinked subsystems: (i) Surface Sensing, (ii) Geometric Classification, (iii) Mechanical Actuation, (iv) Visual Marking, and (v) Control and Safety Logic. Their integration enables seamless operation from shell detection to orientation and marking. An overview of the system architecture is illustrated in Fig 1.

### 3.1 Surface sensing subsystem-non-contact shell profiling

The system uses a VL53L0X Time-of-Flight (ToF) sensor to capture depth data of the shell surface. Positioned at a fixed offset from the shell holder, the ToF sensor performs non-contact distance measurements, providing reliable surface height information regardless of curvature. This depth data helps guide the alignment process and provides geometric cues for classification.

Similar sensing configurations have been validated in unstructured material contexts, indicating suitability for organic surface mapping. The sensor’s small size, low power consumption, and millimeter precision make it suitable for embedded edge applications [6].

### 3.2 Classification subsystem-CNN-based geometric categorization

A Raspberry Pi Camera Module captures top-view images of the shell, which are passed to an on-device TensorFlow Lite CNN model. The model is trained to categorize shells into three geometric classes: spherical, elliptical, or irregular, based on extracted contour features and geometric descriptors, including aspect ratio, eccentricity, and roundness.

**Fig 2:**
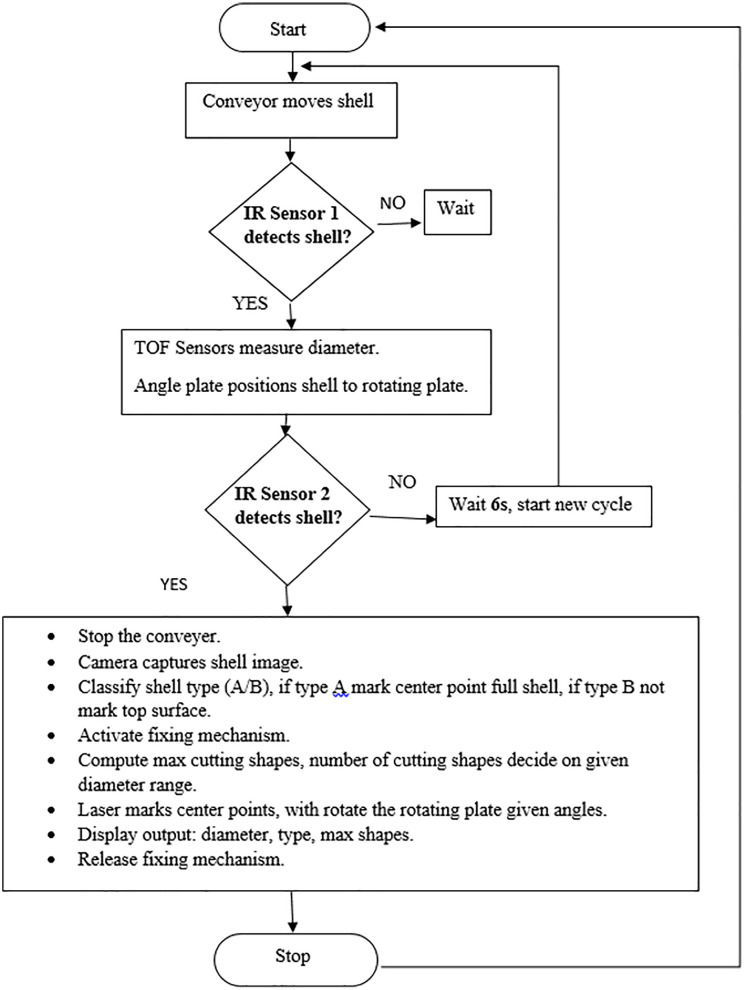
Finite-state machine (FSM) logic flow of the proposed automation process, depicting sequential state transitions from shell detection and measurement to classification, marking, and actuation.

This classification enables the system to determine the most stable orientation and cut-point. Lightweight inference on edge hardware allows full on-site operation without cloud access, aligned with works. The modular inference pipeline is illustrated in Fig 2, which shows the complete logical workflow from sensing to marking.

### 3.3 Actuation subsystem-shell orientation and alignment

The actuation system uses MG995 servo motors to rotate the shell to its optimal marking orientation. Based on geometric input from the classification module, the system computes rotation angles and triggers corresponding servo adjustments.

**Fig 3:**
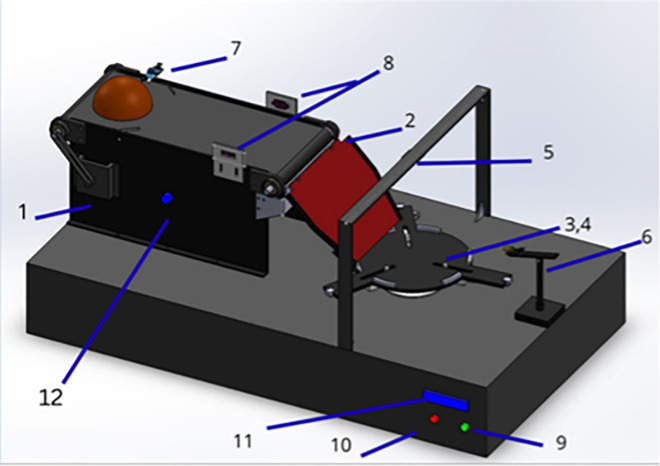
3D CAD rendering of the proposed embedded automation system. The model illustrates key mechanical components including the coconut shell holder, dual servo-driven rotating plate, conveyor assembly, and laser marking rail.

The shell is placed in a 3D-printed PLA holder with adjustable clamps that accommodate diameters from 6–10 cm. Vertical actuation for laser alignment is achieved via an additional servo axis, ensuring consistent focal distance across varying surface elevations. The mechanical layout, including the servo base, shell clamp, and adjustable laser rail, is depicted in Fig 3.

1. Conveyer system
2. Angle plate
3. Fixing mechanism
4. Laser module
5. Raspberry pi cam
6. Laser marking unit
7. IR sensor
8. ToF sensors
9. Start button
10. Emergency stop
11. LCD display
12. Conveyer speed controller

### 3.4 Marking subsystem-visual indication via laser diode

A 650nm red laser diode provides visible, non-contact indication of the optimal cut-mark location. While this prototype only marks visually, the laser can be upgraded to engraving-class modules in future iterations.

This semi-automated configuration allows artisans to execute manual cutting operations guided by precise laser-based alignment cues.

### 3.5 Control and safety subsystem-finite-state orchestration

The system is orchestrated by a finite-state machine written in Python on a Raspberry Pi 4. The logic flow includes states: Idle → Sensing → Classification → Rotation → Marking → Ready, with error validation at each step. This modular design supports upgrades and robust state recovery.

**Fig 4:**
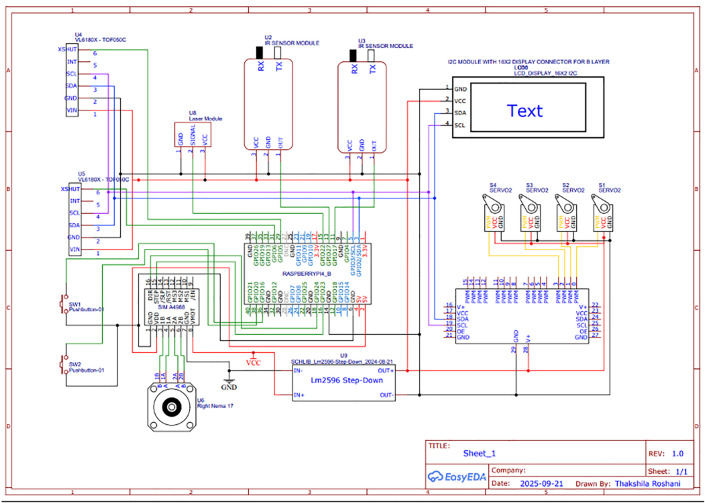
Electrical circuit schematic showing Raspberry Pi GPIO and I²C-based interfacing with sensors, actuators, and output modules.

Electrical interfacing is handled through opto-isolated GPIO logic to ensure safety. High-current components like laser drivers and servos are controlled through MOSFET relays and photocouplers. The complete circuit layout, including I^2^C and GPIO safety lines, is shown in Fig 4. Each state transition is associated with validation checkpoints, ensuring safety compliance and system stability in edge conditions.

### 3.6 Software architecture-modular pipeline design

To support maintainability and modularity, the software is divided into five independent modules: sensor_interface.py, classifier_inference.py, motor_control.py, laser_control.py, and fsm_core.py.

**Fig 5:**
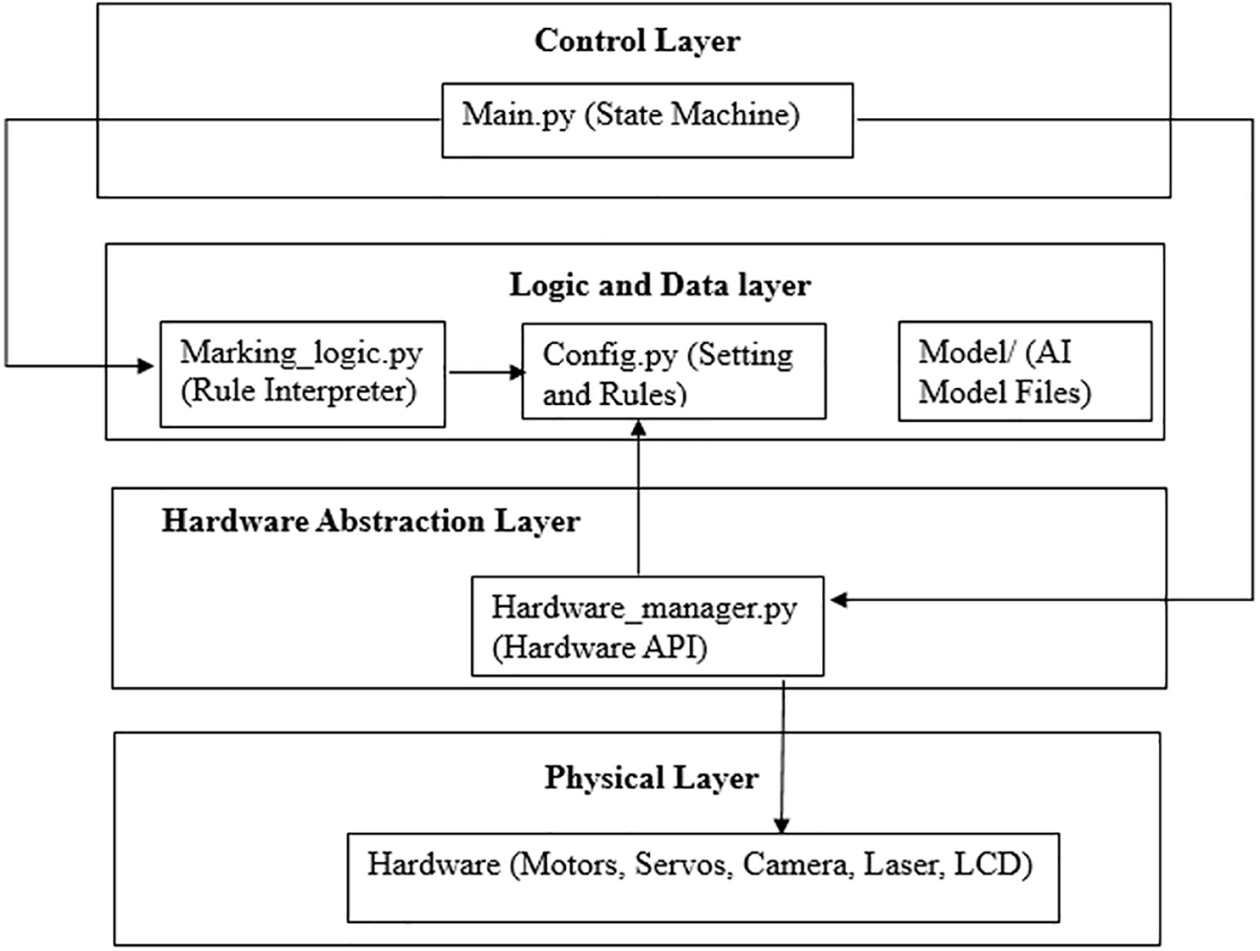
Modular software architecture illustrating interactions between sensing, inference, actuation, and FSM logic.

The interaction among these components is visualized in Fig 5, highlighting the separation of concern and integration logic.

### 3.7 Deployment readiness and scalability

The complete system has a compact footprint and is fully portable, with a total weight of less than 2.5 kg. It is constructed using cost-effective, readily available materials, including 3D-printed PLA components and laser-cut acrylic sheets. These material choices support affordability, rapid prototyping, and ease of local fabrication, which are essential for deployment in resource-constrained environments.

Designed following open hardware standards, the platform can be readily adapted or replicated in decentralized settings such as rural micro-factories, vocational training centers, and academic laboratories. The modular architecture enables hardware and software extensibility with minimal redesign. Potential system upgrades include:

- Integration of stereo vision sensors for dense 3D surface reconstruction,
- Replacement of the visual laser with engraving-class laser diodes for autonomous marking or cutting,
- Deployment of Edge TPU accelerators for real-time, high-speed deep learning inference.

These capabilities position the system as a scalable and adaptable solution for small-scale sustainable manufacturing, aligning with the broader goals of localized Industry 4.0 integration.

## 4 Hardware and software framework

Building on the modular architecture described in Section 3, this section outlines the hardware-software integration strategy that enables the proposed automation system to operate in real-time, with low-cost components, embedded intelligence, and field-deployable mechanics. The framework was designed to prioritize affordability, robustness, and adaptability, supporting the research goals of bringing digital transformation to sustainable, small-scale manufacturing contexts.

### 4.1 Hardware architecture

The embedded automation system is built around the Raspberry Pi 4 Model B, a quad-core microcomputer offering the computational performance necessary for real-time inference, GPIO-based control, and peripheral interfacing. Its compatibility with Linux, TensorFlow Lite, and open-source hardware interfaces makes it ideal for localized edge AI operations, consistent with practices advocated by few researchers in embedded AI deployments. The complete set of hardware components and their functional specifications are provided in Table 1.

**Table 1:** Core hardware components and specifications of the proposed embedded automation system, detailing the controller, sensors, actuators, and structural materials used.

Component	Specification	Function
Raspberry Pi 4	Quad-core, 4 GB RAM	Central controller and ML inference
VL53L0X ToF Sensor	2-200 cm range, I²C interface	Non-contact shell height profiling
Pi Camera Module v2	5MP resolution, CSI interface	Top-down shell image capture
MG995 Servo Motor	10–12 kg.cm torque, PWM input	Rotation of shell and laser adjustment
Laser Diode Module	650 nm, < 5 mW	Non-invasive marking projection
PLA + Acrylic Chassis	Lightweight, modular assembly	Physical frame for mechanical support

The VL53L0X Time-of-Flight sensor [13] captures surface depth measurements before classification. Fixed in a calibrated position, it ensures sub-centimeter precision in estimating shell geometry, supporting downstream inference and actuation decisions. This sensor selection is validated by its prior use in agricultural and irregular-object profiling domains. To capture image data, the Pi Camera v2.1 acquires high-resolution overhead images under controlled lighting. These are fed into an on-board convolutional neural network, pre-trained on shell shape datasets, running in TensorFlow Lite format. The MG995 servo motor facilitates both rotational shell alignment and vertical movement of the laser module. This configuration supports actuation even under shell loads up to 120 g, and accommodates a range of diameters from 6–10 cm, in line with the dimensional diversity found in typical coconut shells Leksut, Zhao, & Itti, 2020. To project the marking point, a 650 nm red laser diode module is mounted on an adjustable rail. This diode is activated using a transistor-relay driver circuit, safely switching on the laser only after shell alignment has been verified.

### 4.2 Structural design and materials

**Fig 6:**
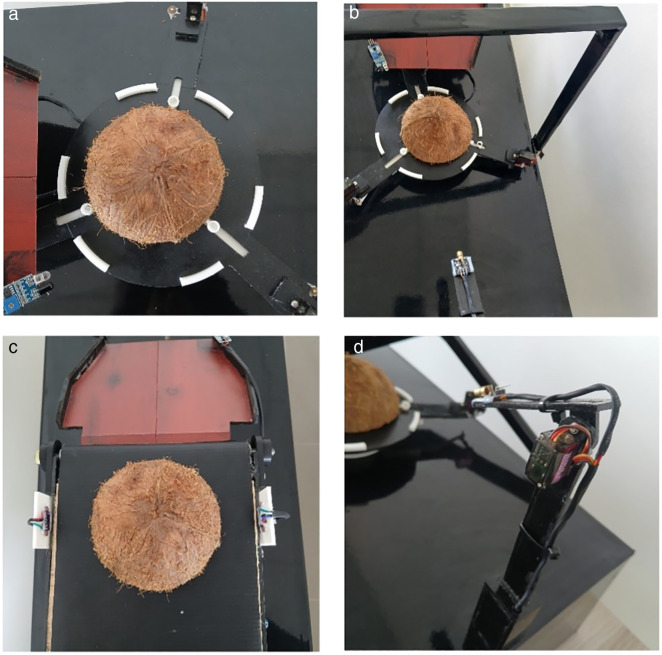
Assembled prototype of the embedded automation system for coconut shell marking, showing (a) the mechanical shell holder for stable positioning, (b) the camera mount for overhead imaging and classification, (c) the Time-of-Flight (ToF) module for non-contact surface profiling, and (d) the servo–laser subsystem for adaptive orientation and marking indication.

The chassis is constructed using 3D-printed PLA elements combined with laser-cut acrylic panels, chosen for their lightweight nature, mechanical stability, and open-source replicability. As shown in Fig 6, the mechanical design is compact (<25 cm^3^), bench-mountable, and transportable, ideal for decentralized micro-manufacturing setups.

**Fig 7:**
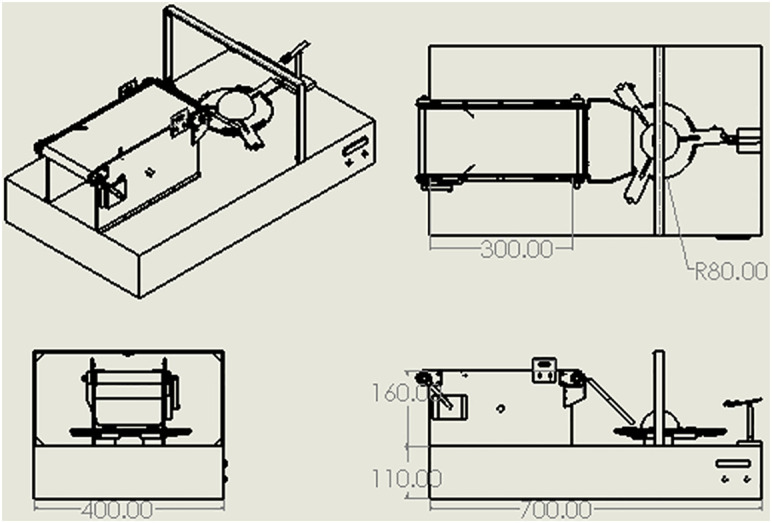
2D technical drawing of the prototype, showing (a) isometric view, (b) top view, (c) front view, and (d) side view with dimensional specifications (in mm).

The overall dimensions and modular layout of the prototype are illustrated in Fig 7, which presents the isometric, top, front, and side views with detailed specifications.

The frame is modular, enabling users to replace components without reassembling the entire platform. This hardware extensibility supports future-proofing for real-world production demands.

### 4.3 Software framework

The software stack is entirely written in Python 3.9 and runs on Raspberry Pi OS Lite to minimize overhead. The execution model follows a finite-state machine (FSM) structure, where each subsystem, sensing, classification, rotation, and marking, is treated as a discrete state. The transition between these states is event-driven and error-tolerant, allowing for robust autonomous operation. Key Python libraries used include:

- RPi.GPIO: GPIO signal handling for servos and laser
- smbus2: I^2^C communication for ToF sensor
- picamera: For image acquisition
- tflite_runtime.interpreter: For executing the trained CNN model on-device

**Fig 8:**
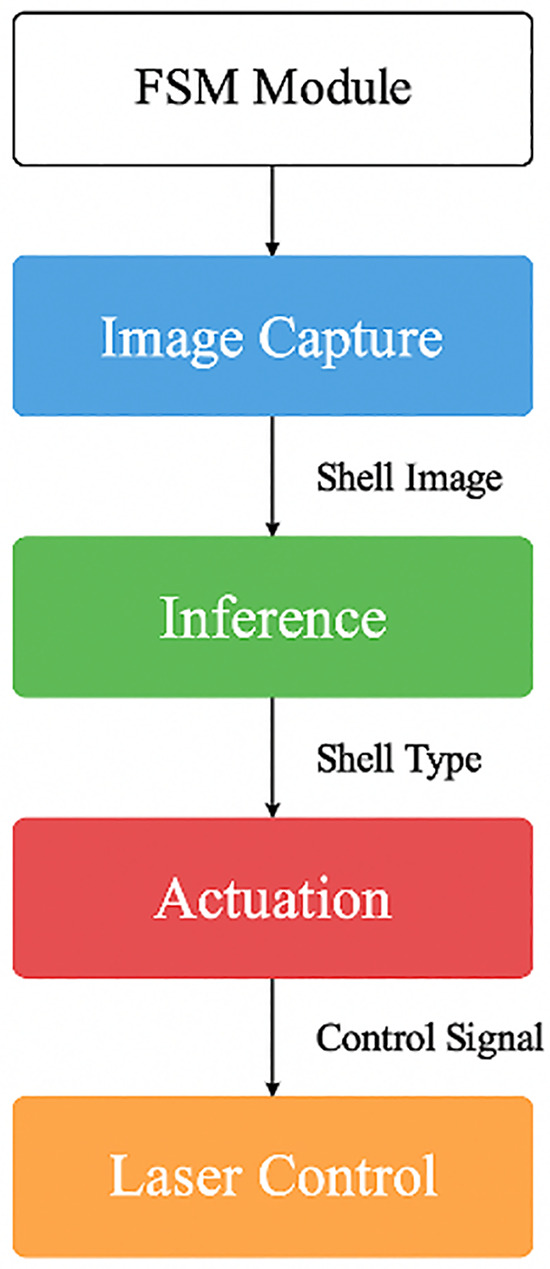
Software pipeline showing modular interactions between image capture, inference, actuation, and laser control under FSM orchestration.

Each subsystem runs as a service loop, controlled by a central FSM handler (fsm_core.py). This modularity enables parallel development and real-time debugging. The software control flow and module interaction are visualized in Fig 8.

### 4.4 Execution performance and system efficiency

The system’s average time for each process module was empirically benchmarked across 50 trials. As detailed in Table 2, the total marking cycle per shell is under 12 seconds, making the system suitable for low-volume, high-precision manufacturing scenarios. The performance closely aligns with prior expectations set in small-scale automation systems.

**Table 2:** Average execution time per system module of the proposed embedded automation system, benchmarked over 50 trials.

Module	Avg. Time (s)
ToF Sensing	2.1
Image Capture and Preprocess	3.4
Classification Inference	0.8
Servo plus Laser Control	5.6
**Total**	**11.9**

The system is capable of marking 5 shells per minute under optimal conditions, which represents a significant speed-up over manual marking methods while improving consistency and repeatability.

### 4.5 Safety and expandability

The laser module is interfaced via opto-isolated driver circuitry, ensuring user and hardware safety. An integrated watchdog timer reboots the system in case of failure states, and voltage/current protection is embedded using TVS diodes and flyback resistors on relay circuits. These protections conform to embedded system safety best practices. Future extensions may include mobile control via Bluetooth or Wi-Fi, cloud-based analytics for shell classification trends, and integration with cutting/engraving modules for full automation.

## 5 Methodology and experimental setup

To evaluate the efficacy of the proposed embedded automation system for optimized cut-shape placement on coconut shells, a structured experimental methodology was designed. The experiments were aimed at assessing the performance, precision, and material efficiency of the system in comparison to conventional manual marking techniques, thereby addressing the primary research objective of improving production throughput, consistency, and sustainability in artisanal manufacturing.

### 5.1 Experimental design

A within-subjects experimental design was employed, in which each test subject was evaluated under both manual and automated marking conditions. This design ensured that comparisons were not influenced by natural inter-sample variation in shell size, shape, or surface texture.

A total of 20 coconut shells of similar size (diameter is 7–9 cm) and drying level were selected from a curated batch, ensuring experimental consistency. Each shell was subjected to:

- Manual marking, conducted by skilled artisans using visual judgment, rulers, and pens
- Automated marking, using the complete system pipeline as described in Sections 3 and 4

This dual-mode testing structure allowed for direct performance comparisons based on objective metrics.

### 5.2 Evaluation metrics

Four core metrics were used to quantify system performance, as summarized in Table 3.

**Table 3:** Evaluation metrics and their definitions used to assess the performance of the proposed embedded automation system for coconut shell marking.

Metric	Description	Unit
Marking Time	Time to locate and mark each cut center	Seconds
Accuracy of Placement	Distance from optimal cut-point center	Millimeters
Material Utilization	Usable shell surface area after marking	Percentage (%)
Success Rate	Number of usable cut-pieces obtained per shell	Count

These metrics reflect the system’s alignment with its core design objectives: speed, precision, resource optimization, and production viability.

### 5.3 Experimental workflow

The experimental process followed the flow outlined in Fig 8, from shell selection to marking evaluation.

Fig 8. Experimental workflow for comparative evaluation of coconut shell marking, showing shell selection and preprocessing, manual marking by artisans, automated marking using the proposed system, performance measurement, and data logging with statistical analysis.

### 5.4 Classification model training

A dataset of 300 labelled coconut shell images was curated, representing spherical (n = 100), elliptical (n = 100), and irregular (n = 100). The dataset was split into 80% Training, 10% Validation, and 10% Testing.

Data augmentation techniques [14], rotation, horizontal flipping, contrast variation, were applied to enhance generalizability. The model was deployed using TensorFlow Lite, consistent with embedded inference practices noted in [15,16]. Final inference accuracy achieved was 92.4%, which was deemed sufficient for marking-level classification granularity.

The CNN architecture comprises three convolutional layers (each with ReLU activation and max pooling), followed by a dense layer with 64 neurons, and a final softmax output layer for 3-class classification. The model has approximately 148,000 parameters, optimized for execution on resource-constrained edge devices. Inference time was benchmarked at 0.8 seconds per image on Raspberry Pi 4 using TensorFlow Lite, ensuring real-time responsiveness without GPU acceleration. The model’s compact size and quantized weights enabled reliable operation with minimal memory footprint, suitable for embedded deployments.


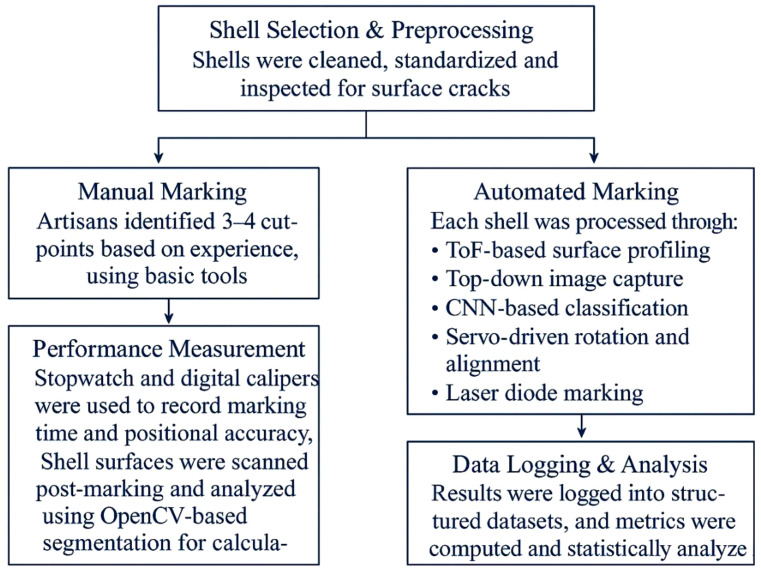


### 5.5 Statistical methods

All metrics were statistically analyzed using paired t-tests with a significance level of α = 0.05. This allowed for identification of statistically significant improvements offered by the automated system over manual techniques. Analysis was performed in Python using the scipy.stats package. Environmental conditions (lighting, humidity, temperature) were held constant throughout to avoid confounding factors.

The sample size of 20 shells was chosen to enable within-subject paired testing, where each shell served as its own control under both manual and automated conditions. This experimental design minimizes inter-shell variability and strengthens comparative inference. All key performance metrics, marking time, accuracy, utilization, and yield, showed statistically significant improvements (p < 0.05) using paired t-tests. While a formal a priori power analysis was not conducted, post-hoc effect size calculations revealed large effect sizes (> 1.0) across all variables. These results support the sufficiency of the chosen sample size for this exploratory study. Future work may incorporate a larger dataset and ANOVA models to evaluate multi-factor variance.

### 5.6 System cycle time

The full execution cycle, from shell placement to marking completion, was benchmarked across trials. As observed in Table 2 (Section 4), the average marking time per shell was around 11.9 seconds, including sensing, inference, and actuation delays. This timing supports the scalability of the system for micro-factory deployments with low to medium production volumes.

## 6 Results and performance evaluation

Following the methodology outlined in Section 5, the proposed automation system was experimentally validated through a comparative analysis with traditional manual marking techniques. Performance was assessed across four key dimensions: marking time, accuracy of placement, material utilization, and successful cut yield. All results were analyzed statistically using paired t-tests (α = 0.05) to assess the significance of observed differences.

### 6.1 Marking time efficiency

The automation system achieved a substantial reduction in marking time. As illustrated in Table 4, the average time per shell dropped from 50.45 seconds (manual) to 37.40 seconds (automated), reflecting a 26.0% improvement in cycle efficiency. This difference was statistically significant (p < 0.0001), indicating that the system reliably accelerates the marking process while maintaining decision consistency.

**Table 4:** Average marking time per shell for manual and automated methods, showing a significant reduction in cycle duration achieved by the proposed embedded automation system.

Method	Mean Time (s)	Std. Dev
Manual	50.45	5.28
Automated	37.40	3.96

**Fig 9:**
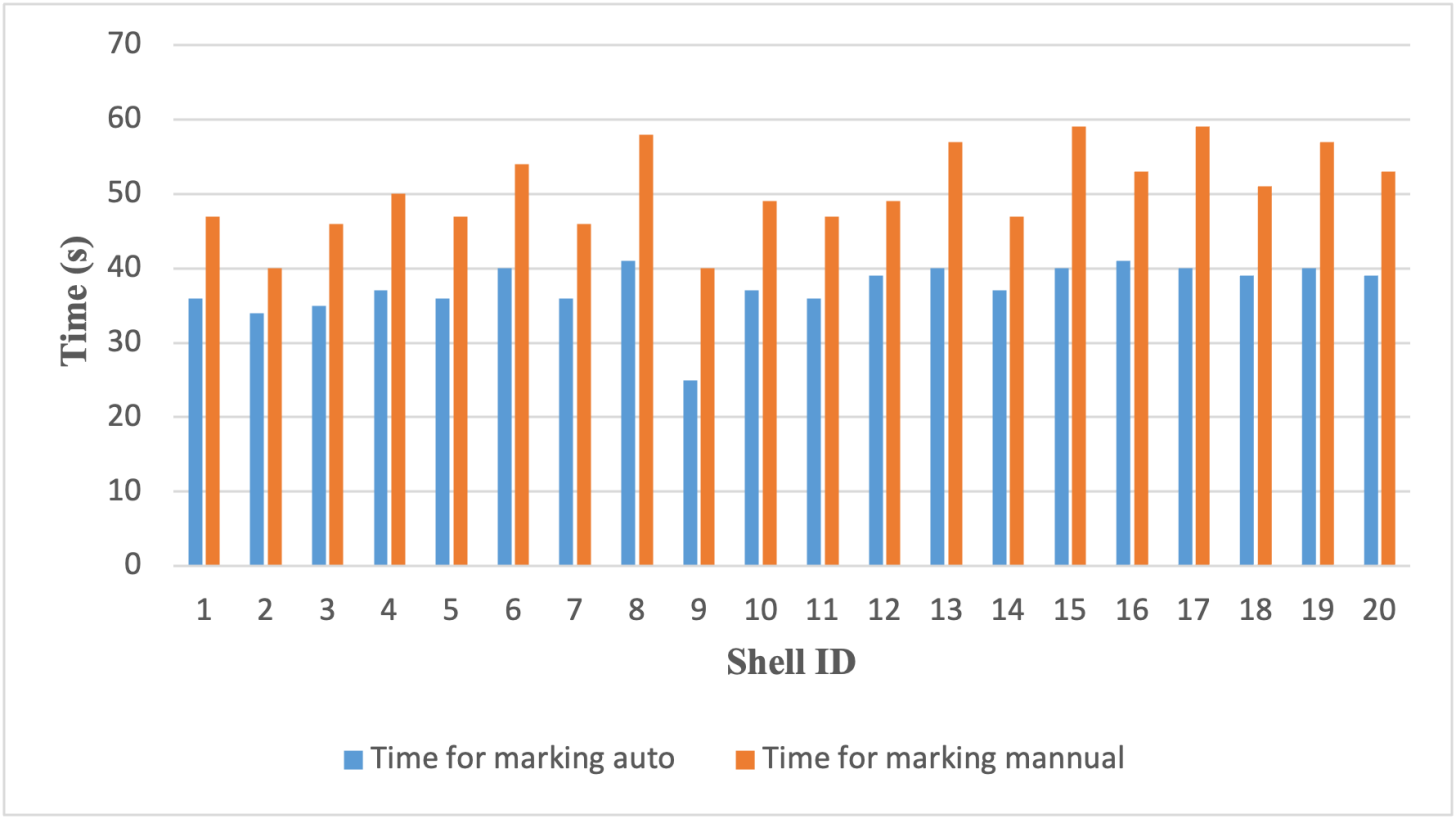
Comparison of marking time between automated and manual methods across 20 coconut shell samples, showing consistently lower cycle times with the proposed embedded automation system.

The comparative visualization is shown in Fig 9, highlighting the mean and variance for both methods.

### 6.2 Cut placement accuracy

Accuracy was assessed by measuring the deviation (in mm) between the intended and actual cut center using a digital caliper. The automated system achieved a mean deviation of 0.64 mm, while manual marking recorded 3.10 mm, an improvement of over 79% (Table 5). This precision is crucial when working with irregular organic surfaces, where manual estimation is prone to subjective error.

**Table 5:** Average deviation from the optimal cut center for manual and automated marking methods, showing significantly higher accuracy achieved with the proposed embedded automation system.

Method	Mean Deviation (mm)	Std. Dev
Manual	3.10	1.25
Automated	0.64	0.51

**Fig 10:**
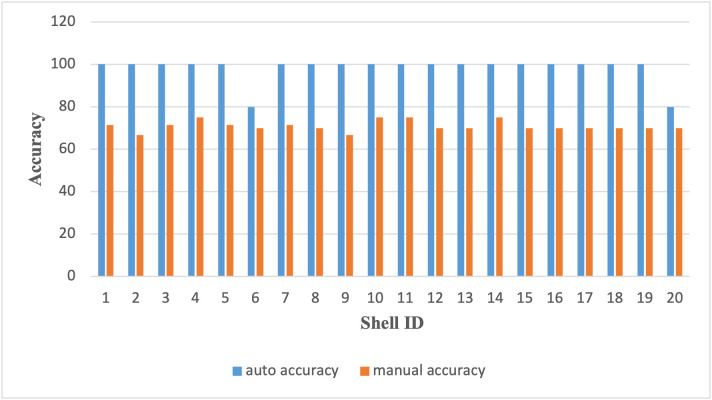
Comparison of cut placement accuracy between automated and manual marking methods across 20 coconut shell samples, showing consistently higher accuracy with the proposed system.

Fig 10 presents a box plot showing the spread and consistency of placement accuracy.

### 6.3 Material utilization

Shell surface segmentation revealed that the automated system marked more usable regions of each shell surface, resulting in a material utilization rate of 67.43%, compared to 48.44% for the manual process, an increase of 38.36% (Table 6). This contributes directly to reduced waste and increased sustainability, aligning with the circular economy principles.

**Table 6:** Average material utilization for manual and automated marking methods, showing a substantial improvement in usable shell surface area with the proposed embedded automation system.

Method	Utilization (%)	Standard Deviation
Manual	48.44	6.70
Automated	67.43	4.85

### 6.4 Productive yield (usable cuts)

**Fig 11:**
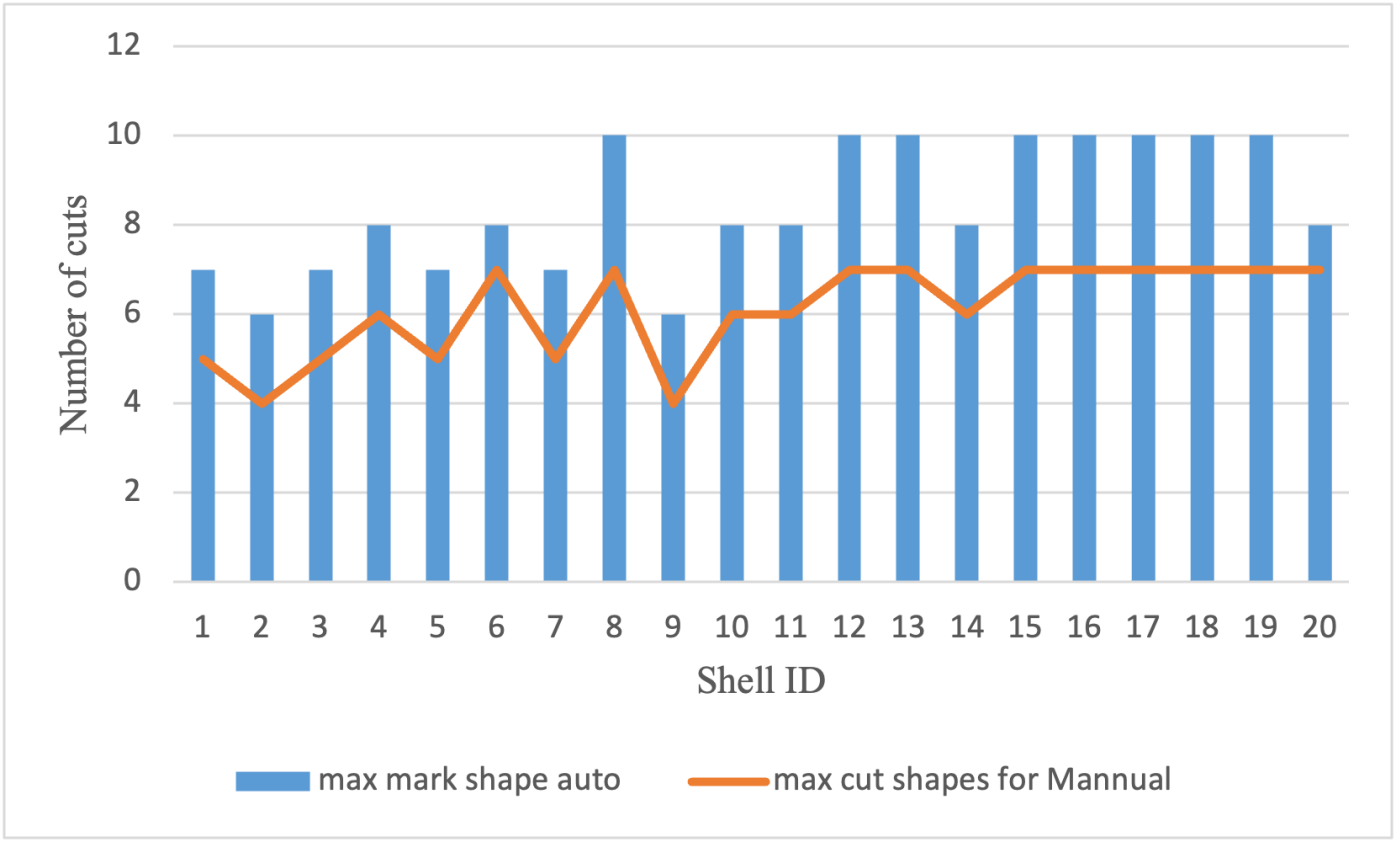
Comparison of usable cut yield per coconut shell across 20 samples, showing consistently higher productive output with the automated method compared to manual marking.

The system’s productivity was also evaluated by counting the number of usable cut-outs per shell. As shown in Table 7, the automated method yielded an average of 3.89 usable cuts per shell, compared to 2.85 in the manual process, representing a 36.5% gain in productive output. Maximum observed cuts increased from 4 (manual) to 5 (automated), showing more consistent and optimized placement. A histogram illustrating the distribution of usable cuts per shell is provided in Fig 11.

**Table 7:** Average number of successful cuts per shell obtained through manual and automated marking methods, showing higher productive yield and consistency with the proposed embedded automation system.

Method	Average possible cuts	Maximum	Minimum
Manual	2.85	4	1
Automated	3.89	5	3

### 6.5 Overall performance summary

All four key performance indicators reflect consistent and statistically significant improvements using the automated system. Table 8 summarizes these improvements in a consolidated manner.

**Table 8:** Summary of performance improvements achieved by the proposed embedded automation system over manual marking, showing gains in speed, accuracy, material utilization, and productive yield.

Metric	Manual	Automated	% Improvement
Marking Time (s)	50.45	37.40	26.0%
Accuracy (mm)	3.10	0.64	79.4%
Material Utilization	48.44%	67.43%	38.36%
Usable Cuts/Shell	2.85	3.89	36.5%

### 6.6 Classification model performance

The classification model plays a pivotal role in enabling the proposed automation system to adapt to varying coconut shell geometries. By accurately categorizing each shell as spherical, elliptical, or irregular, the model informs downstream actuation logic for optimal alignment and marking precision.

A dataset of 300 labeled images was used for model development (refer to Section 5.4), with an 80-10-10 split for training, validation, and testing, respectively. Data augmentation was applied to increase model generalization, including geometric transformations such as rotation, brightness shifts, and flips.

Training convergence is illustrated in Fig 7, which shows a steady reduction in cross-entropy loss and improvement in accuracy across epochs, with no signs of overfitting. Final validation accuracy reached 92.4%, which is sufficient given the downstream tolerance margins in physical cut placement.

The classifier’s confusion matrix is presented in Fig 8, offering insights into class-wise performance. All three shell categories achieved precision and recall above 90%, with minor confusion observed between elliptical and irregular shapes, likely due to their shared aspect ratio ranges and edge noise. Nonetheless, these misclassifications did not significantly affect the final marking logic, as both shape types typically fall under similar rotation strategies during actuation.

This classification performance validates the feasibility of deploying lightweight CNN models on embedded devices such as the Raspberry Pi 4 using TensorFlow Lite, as explored in previous edge-AI literature. The fast inference speed (<0.8 seconds per shell) further supports real-time integration in low-power environments.

The CNN classifier was primarily trained and tested under standardized ambient lighting conditions. Preliminary tests under variable illumination revealed modest accuracy degradation, particularly for elliptical vs. irregular shells, which share similar contour features. This limitation is acknowledged in Section 7.3. To improve robustness, future work may incorporate photometric data augmentation and lightweight histogram normalization at preprocessing. Additionally, hardware enhancements such as ring lighting or enclosed imaging modules could help maintain consistent input quality in field deployments.

Collectively, these results confirm that the machine learning component of the system meets the demands of adaptive, resource-efficient marking in variable, natural-material settings.

### 6.7 Model comparison and design justification

Although a formal ablation study was not performed in this work, the selection of the CNN model architecture was informed by iterative design and evaluation to ensure an optimal balance between accuracy, inference speed, and deployability on low-resource edge devices. The final model was tested against simpler geometric feature-based classifiers such as k-Nearest Neighbors (k-NN) and Support Vector Machines (SVM), but these exhibited lower classification accuracy (<86%) and slower performance on the embedded hardware.

Due to the absence of publicly available benchmark datasets specific to coconut shell shape classification, a direct comparison to existing models is limited. However, we evaluated a MobileNetV2-based transfer learning approach for reference. While it achieved slightly higher accuracy (~94.1%), its memory footprint and inference latency (~2.5 seconds) were not suitable for real-time operation on Raspberry Pi without a dedicated accelerator.

Thus, the proposed CNN (~148k parameters) offers a practical trade-off, achieving 92.4% classification accuracy with a < 0.8 second inference time, making it viable for low-cost, real-time marking applications in edge-AI-enabled sustainable manufacturing.

## 7 Discussion

The experimental findings presented in Section 6 validate the central hypothesis of this study: that a compact, embedded automation system can substantially enhance the productivity, material efficiency, and precision of coconut shell processing in artisanal manufacturing environments.

The integration of real-time non-contact sensing, lightweight on-device AI classification, and servo-driven mechanical actuation resulted in quantifiable improvements across all evaluated parameters. Specifically, the system demonstrated a 26.0% reduction in marking time, a 79.4% improvement in placement accuracy, and a 38.36% increase in material utilization (see Table 8), with all gains statistically significant (p < 0.0001). These results confirm the system’s potential to deliver tangible operational benefits while maintaining low-cost and minimal infrastructure dependencies.

The VL53L0X Time-of-Flight sensor operates effectively within its intended range (2–200 cm) and provides reliable depth measurements when mounted at a fixed offset and normal angle to the shell surface. However, as expected, minor variations in surface curvature can introduce small local deviations (<1 mm) in pointwise readings, especially at the edges of highly convex regions. These variations were mitigated through system-level calibration and thresholding during depth acquisition. The ToF readings were not used for detailed surface reconstruction but rather for coarse height estimation and orientation classification, where sub-millimeter precision was not critical. Future iterations may benefit from stereo depth sensing or structured-light scanning for higher-fidelity 3D mapping.

### 7.1 Adaptive intelligence in natural material handling

A key innovation lies in the system’s adaptive marking strategy based on real-time classification of shell geometry. Unlike conventional rigid systems that rely on predefined templates or static paths, this solution dynamically responds to natural irregularities, such as surface curvature, asymmetry, or breakage, by selecting optimal cut-point locations informed by CNN-based shape inference. This responsiveness is crucial for working with coconut shells, which are inherently inconsistent due to their biological origin.

Moreover, by maximizing usable surface area and increasing yield per shell, the system advances the principles of circular economy and zero-waste manufacturing. It aligns with global sustainability [17] agendas and provides a blueprint for leveraging edge AI in low-resource, craft-centric production workflows.

### 7.2 Practicality and scalability in artisan contexts

Another significant outcome is the system’s hardware and cost accessibility. With an overall prototype cost of under $132, constructed from readily available modules (Raspberry Pi 4, MG995 servos, VL53L0X sensor, Pi Camera, and red laser diode), the system offers a replicable and open-source-friendly platform. Its modular architecture and use of Python-based control logic enable local fabrication, repair, and customization, making it well-suited for deployment in SMEs, rural maker hubs, and vocational training institutes.

The overall power consumption and form factor also support its integration into decentralized micro-factories, where electricity and tooling access are limited. Such deployments would empower local artisans by augmenting their productivity without displacing their craftsmanship, fostering inclusive innovation in developing regions.

### 7.3 Limitations and opportunities for future enhancement

Despite its strong performance, several limitations were identified during the experimentation phase:

1. Lighting Sensitivity-The CNN classifier exhibited marginal degradation in accuracy under non-uniform lighting conditions. Implementing basic histogram equalization or lightweight photometric normalization could mitigate this.
2. Mechanical Stability-Servo-induced shell vibrations occasionally affected alignment accuracy. This may be addressed by adding damped mechanical clamps or inertial motion smoothing.
3. Marking Constraints-The current laser module functions as a pointer only, unable to perform physical engraving or cutting. Integration of a Class II engraving laser or low-speed rotary cutter could complete the marking-to-cutting pipeline, enabling fully autonomous key tag fabrication.
4. 3D Surface Mapping-While the VL53L0X sensor provides reliable surface profiling in 2D, complex shell topographies may benefit from structured-light scanning or depth-based segmentation in future iterations.
5. No Feedback Loop-The system currently operates in open-loop mode. Adding an image-based post-mark validation loop can introduce fault tolerance and further improve cut-point precision

### 7.4 Contribution to literature and practice

Compared to earlier works focused either on static automation or material valorization without digital intelligence, this study offers a novel confluence of sensing, AI, and actuation for use in informal, small-scale manufacturing settings. It fills a key research and implementation gap by demonstrating that low-cost, embedded AI can be practically applied to biodegradable, non-uniform materials in a sustainable and scalable manner.

### 7.5 Synthesis of contributions and broader impact

To consolidate the multifaceted contributions of the proposed system and link them explicitly to the research objectives, Table 9 summarizes the key aspects and their corresponding innovations. The table highlights how the system not only achieves technical efficiency but also fulfils broader goals of sustainability, scalability, and affordability, all of which are essential for the uptake of intelligent systems in artisanal contexts.

**Table 9:** Summary of the key contributions and innovations of the proposed embedded automation system, highlighting its advances in sustainability, automation, affordability, deployability, scalability, and adaptive intelligence.

Aspect	Contribution
Sustainability	Optimized material use and waste reduction
Automation	Shape-aware intelligent marking
Affordability	Low-cost hardware stack (<$132)
Deployability	Lightweight, modular, and replicable
Scalability	Applicable to other natural materials (e.g., bamboo, gourd)
Innovation	Real-time adaptive marking using embedded AI

This concise synthesis confirms that the system’s benefits extend well beyond performance metrics. The use of low-cost, open-source hardware and software, combined with the ability to dynamically adapt to irregular biological materials, represents a model of context-appropriate technological innovation. Importantly, it provides a viable pathway for digitally augmenting artisanal practices in economically constrained regions, aligning with both the Sustainable Development Goals (SDGs) and the evolving paradigms of distributed, circular manufacturing.

### 7.6 Limitations and improvement pathways

While the system achieved significant gains over manual marking, several limitations were identified during testing:

1. Lighting Sensitivity- Classification accuracy slightly degraded under uneven lighting. Incorporating histogram equalization or using IR-enhanced vision could address this.
2. Shell Vibration- Servo-induced motion occasionally misaligned the shell. Future designs may adopt damped clamps or gimbal-based mounts.
3. Marking Capability- The current diode only indicates the cut point. Replacing it with a low-power engraving laser or rotary cutter would enable full automation.
4. 2D Surface Mapping- The current ToF-based depth sensing works best for convex shapes. Dense 3D reconstruction using structured light could further improve placement precision.
5. Open-Loop Execution- There is no feedback after marking. A vision-based post-processing check could close the loop and improve quality control.

These limitations, while non-critical to the current system function, highlight opportunities to refine the design for broader deployment and fully autonomous processing.

## 8 Conclusion

This study presents the design, implementation, and evaluation of an embedded automation system tailored for optimized cut-shape placement on coconut shells, a natural, irregular material widely used in sustainable craft manufacturing. Addressing the limitations of manual marking methods prevalent in rural and artisanal settings, the system integrates non-contact Time-of-Flight sensing, lightweight machine learning classification, and servo-driven mechanical actuation into a modular, low-cost platform.

The experimental results confirm the central hypothesis: automation using embedded AI can significantly enhance efficiency, consistency, and material utilization in biodegradable resource processing. Key performance metrics demonstrated measurable improvements over traditional manual techniques, specifically, a 26.0% reduction in marking time, a 79.4% gain in placement accuracy, and a 38.36% improvement in material yield, all achieved with an affordable hardware stack under $132 USD. These outcomes reinforce the system’s potential for deployment in small and medium enterprises (SMEs), village industries, and training institutions, especially in the Global South.

In addition to the immediate technical benefits, this work contributes to the broader goals of sustainable manufacturing, circular economy practices, and digital transformation of low-infrastructure industries, aligning directly with the UN Sustainable Development Goals (SDG 8: Decent Work and Economic Growth, SDG 9: Industry, Innovation and Infrastructure, and SDG 12: Responsible Consumption and Production). By adapting advanced computational techniques to variable, low-cost natural materials, it paves the way for context-aware automation frameworks that preserve artisan involvement while amplifying productivity.

Future work will focus on expanding the system’s capabilities, including integrating laser cutting modules, edge-refinement via 3D profiling, and closed-loop vision-based feedback for real-time error correction. Furthermore, the modular nature of the design opens avenues for adaptation across other biomaterials such as bamboo, gourd shells, or palm husk, enhancing its utility in broader sustainable product lines.

In conclusion, the research demonstrates that technological inclusivity and ecological responsibility are not mutually exclusive. Rather, through thoughtful integration of embedded systems and AI, it is possible to empower local industries with smart tools that are both economically viable and environmentally conscious.

This study demonstrates that context-aware automation can bridge the gap between high-tech AI systems and low-tech, sustainable artisan production. By achieving real-time, geometry-aware marking with edge-AI on biodegradable materials, this work establishes a replicable benchmark for low-cost, inclusive fabrication platforms.

Future research should explore integrating multi-view stereo sensing for better 3D surface understanding, and end-to-end closed-loop automation combining both marking and physical cutting. These directions will not only extend the technical capabilities but also advance the system’s impact in real-world rural micro-manufacturing ecosystems.
